# Supplementary material for: Knowledge-driven design of solid-electrolyte interphases on lithium metal via multiscale modelling
Source: Nat Commun. 2023 Oct 26;14:6823. doi: 10.1038/s41467-023-42212-7 (PMC10603056; doi:10.1038/s41467-023-42212-7)
Supplement: Supplementary file 1 — Supplementary information [file 41467_2023_42212_MOESM1_ESM.pdf]

Supplementary Information

Knowledge-driven design of Solid-Electrolyte Interphases on lithium metal via multiscale modelling

Janika Wagner-Henke[1], Dacheng Kuai[2,3], Michail Gerasimov[1], Fridolin Röder[4], Perla B. Balbuena[2,3,5], Ulrike Krewer[1]*

[1]Institute for Applied Materials – Electrochemical Technologies, Karlsruhe Institute of Technology, Karlsruhe 76131, Germany

[2]Department of Chemical Engineering, Texas A&M University, College Station, Texas 77843, United States

[3]Department of Chemistry, Texas A&M University, College Station, Texas 77843, United States

[4]Bavarian Center for Battery Technology (BayBatt), University of Bayreuth, Bayreuth 95448, Germany

[5]Department of Materials Science and Engineering, Texas A&M University, College Station, Texas 77843, United States

Content

1. Model parameters 3
2. kMC algorithm 4
3. Derivation of electron factor $\boldsymbol{\sigma}$ 4
4. Implemented reaction network 5
5. Comparison of kMC and literature-based MD results 5
6. Further simulation results 6
7. Effect of the electrical double layer on the SEI formation in our model: 8
8. Details on DFT results 9

1. Model parameters

Supplementary Table 1 List of further model parameters

Parameter	Description	Value	Unit	Source
β	Symmetry factor	0.5	[-]	Chosen
k_0	Frequency factor	10^{13}	[-]	Chosen according to literature[1]
D	Diffusion coefficient	$2.27 \cdot 10^{-10}$	$\frac{\mathrm{m}^2}{\mathrm{s}}$	Chosen according to literature[2,3]
ΔL	Distance of surface sites	$3.443 \cdot 10^{-10}$	m	Literature value[2]
C^{DL}	Double layer capacitance	0.2	$\frac{\mathrm{F}}{\mathrm{m}^2}$	Chosen according to literature[4]
T	Temperature	298.15	K	In accordance with DFT calculations
p_{el}	Electron probability at maximum electron transport distance	1	%	Chosen
$z_{Li,max}$	Maximum height of lithium layers	$10.33 \cdot 10^{-9}$	m	Chosen
$z_{el,max}$	Maximum electron transport distance	$2 \cdot 10^{-9}$	m	Literature value[5]
R	Ideal gas constant	8.314	$\frac{\mathrm{J}}{\mathrm{mol\ K}}$	-
F	Faraday constant	$9.648 \cdot 10^{4}$	$\frac{\mathrm{C}}{\mathrm{mol}}$	-
k_B	Boltzmann factor	$1.38 \cdot 10^{-23}$	$\frac{\mathrm{J}}{\mathrm{K}}$	-
N_A	Avogadro constant	$6.022 \cdot 10^{23}$	$\frac{1}{\mathrm{mol}}$	-
E_A^{bond}	Binding energy lithium metal	436.8	kJ/mol	*
n_x	Number of sites in x-direction	15	[-]	Chosen
n_y	Number of sites in y-direction	15	[-]	Chosen
n_z	Number of sites in z-direction	75	[-]	Chosen
ϵ_R	Relative permittivity of EC	90.36	[-]	Literature value[6]

* The binding energy is chosen in such a way that the oxidation probability of a lithium atom embedded in a smooth surface is 5% compared to a lithium metal atom without lithium neighbors.

2. kMC algorithm

Variable Step Size combined with structured lists following[7]:

1. Precalculation of all possible processes and transition rates and set up of structured lists: event list $L_{n,k}$, address list A_m, rate list R_n, counter list C_n
2. Calculation of process probabilities $\Gamma_n = R_n \cdot C_n$ and overall rate $\Gamma_{total} = \sum_n \Gamma_n$
3. Drawing first random number $\zeta_1 \in (0, 1]$ and calculation of time step $\Delta t_i = -\frac{\ln(1-\zeta_1)}{\Gamma_i^{tot}}$
4. Drawing of second random number $\zeta_2 \in (0,\ \Gamma_{total}]$ and selection of process n and site k
5. Perform chosen event
6. Local update of structured lists for all sites included in the performed process and the related next-neighbouring sites
7. If total simulated time $t_{KMC} < t_{end}$ go back to 2

3. Derivation of electron factor *σ*

- Probability of electrons is 1 in and directly on lithium metal surface. This leads to $z = 1, for\ z \leq z_{Li,max} + \Delta L$
- There is no electron transport above the maximum electron transport distance $z_{el,max}$. Therefore holds: $z = 0, for\ z \geq z_{Li,max} + \Delta L + z_{el,max}$
- The electron probability at the maximum electron transport distance $z_{el,max}$ is p_{el}. An exponential decay in the form of $\sigma(z) = \exp(-\beta \cdot \Delta z)$ of the electron probability is assumed between the lithium metal surface and the maximum electron transport distance. Substituting p_{el} and $z_{el,max}$ into this equation gives: $p_{el} = \exp(-\beta \cdot z_{el,max})$. Rearrangement leads to $\beta = \frac{\ln(p_{el})}{z_{el,max}}$. Substituting this into the exponential decay equation yields $\sigma(z) = \exp(\frac{\ln(p_{el})}{z_{el,max}} \cdot \Delta z)$.

4. Implemented reaction network

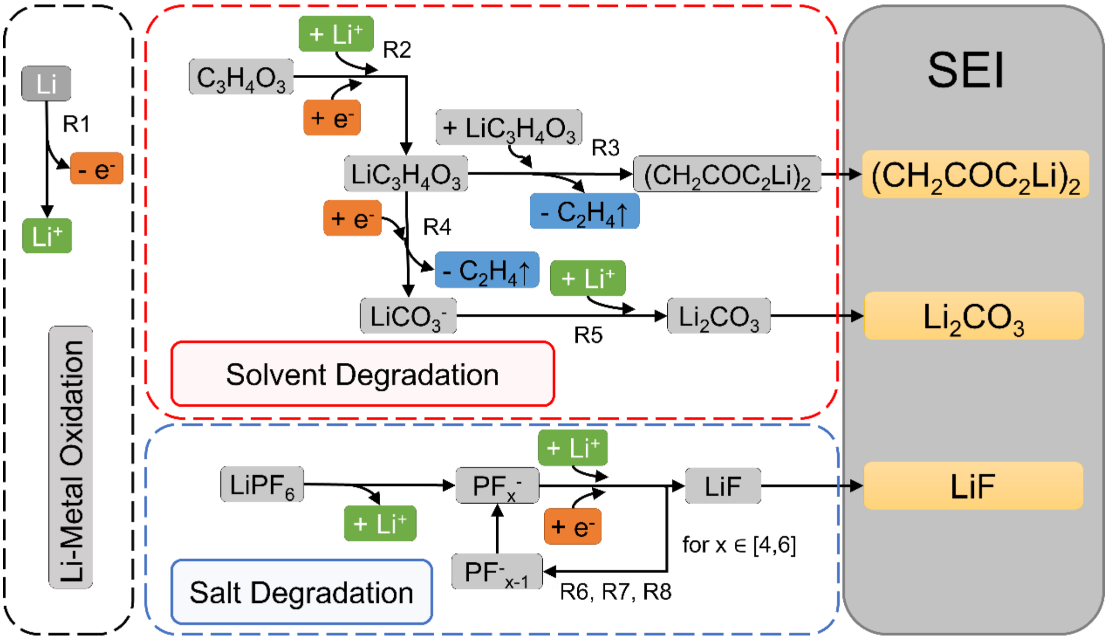

Supplementary Figure 1 Reaction network considered in the multiscale kMC model. It consists of lithium metal oxidation, solvent degradation and salt degradation. Full equations of reactions R1 – R8 are given in Table 1 of the main manuscript.

5. Comparison of kMC and literature-based MD results

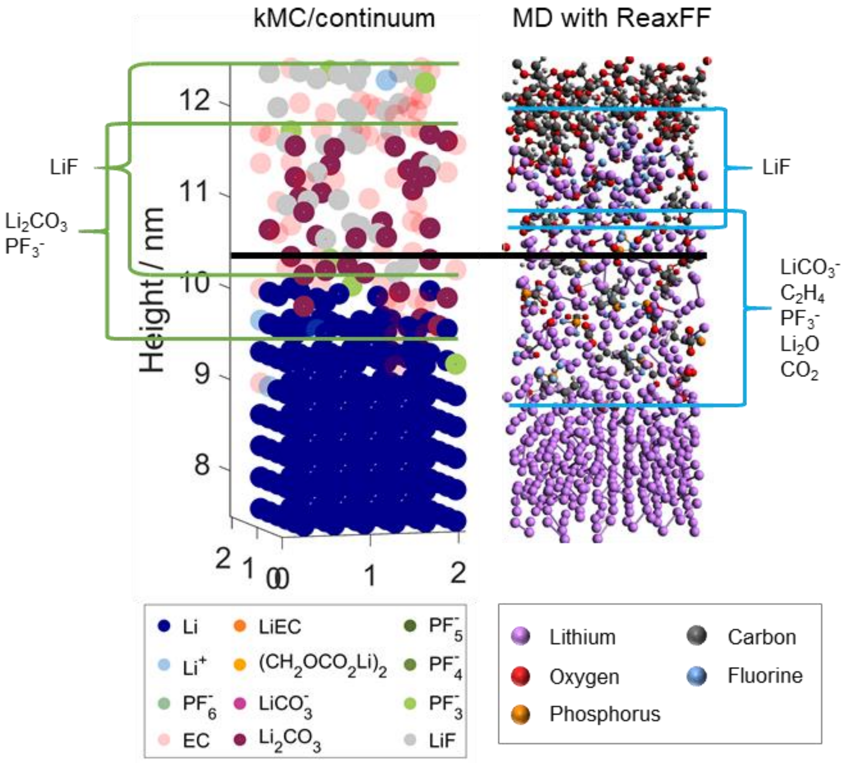

Supplementary Figure 2 Comparison of SEI structure in EC + $LiPF_6$ from kMC-continuum for a ring-opening energy of 12.05 kcal/mol (left) and *ReaxFF* MD (right) after 20 ns. The dimensions of both boxes are 2.07 nm x 2.07 nm x 4.96 nm, each. The kMC result is taken as a representative sample from the large-scale simulations of 5.165 nm x 5.165 nm x 25.823 nm. The MD simulations were provided by Ospina-Acevedo et al.[8].

6. Further simulation results

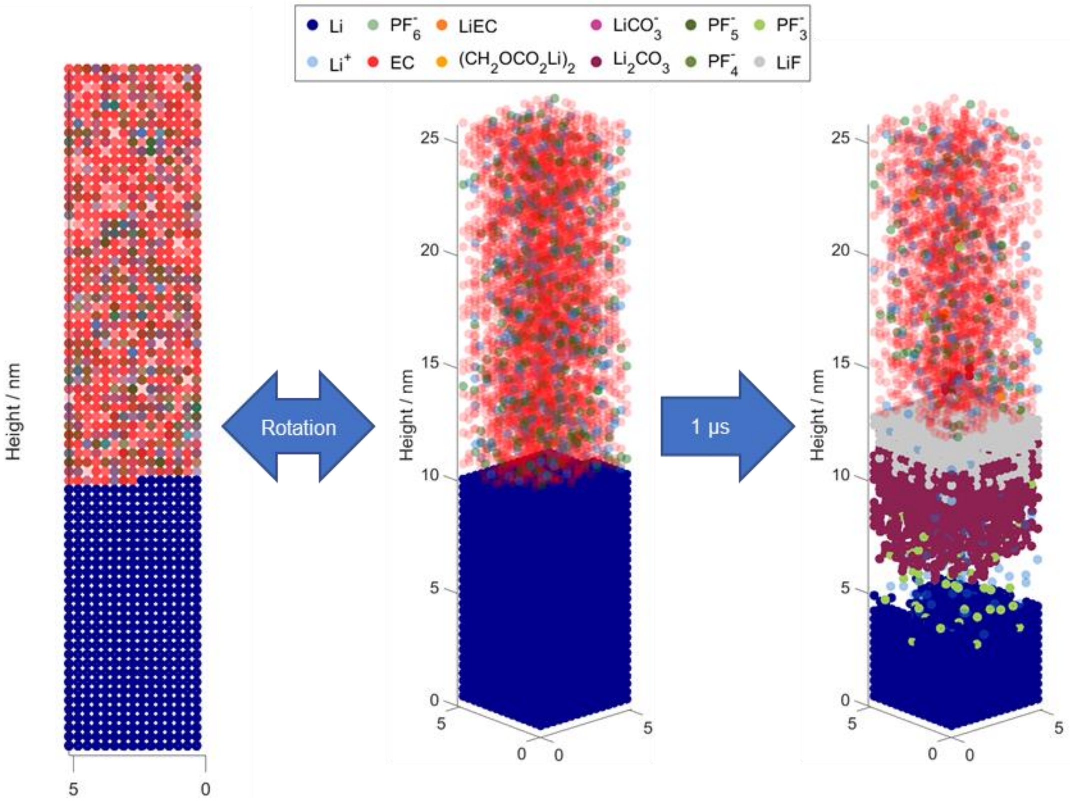

Supplementary Figure 3 SEI formation on a non-uniform lithium metal surface with an implemented step in the initial lithium metal crystal structure. Left: Initial kMC-box with step in the crystal structure in 2D. Center: Initial kMC-box with step in the crystal structure rotated in 3D. Right: Final kMC-box showing the SEI structure after 1µs of simulated time. Source data are provided as a Source Data file.

In order to evaluate the effect of smaller inhomogeneities of the lithium metal surface morphology on the initial SEI formation, we introduced a monolayer step in the initial lithium metal crystal structure and ran the simulation with the same parameters which were used for the results in Figure 3 and Figure 4. The comparison between the final configuration after 1µs with the monolayer step in Supplementary Figure 3 and without the monolayer step in Figure 3 demonstrates that there is – apart from stochastic variations – no significant difference in the formed SEI. In both cases, a Li_2CO_3-layer forms below the initial surface with a denser and thinner LiF-layer above. Moreover, the overall thickness of the initial SEI remains the same. From this we conclude, that there is no major impact of small surface inhomogeneities on the investigated scale on the formed SEI within the first microsecond after the initial contact between the liquid electrolyte and the lithium metal anode.

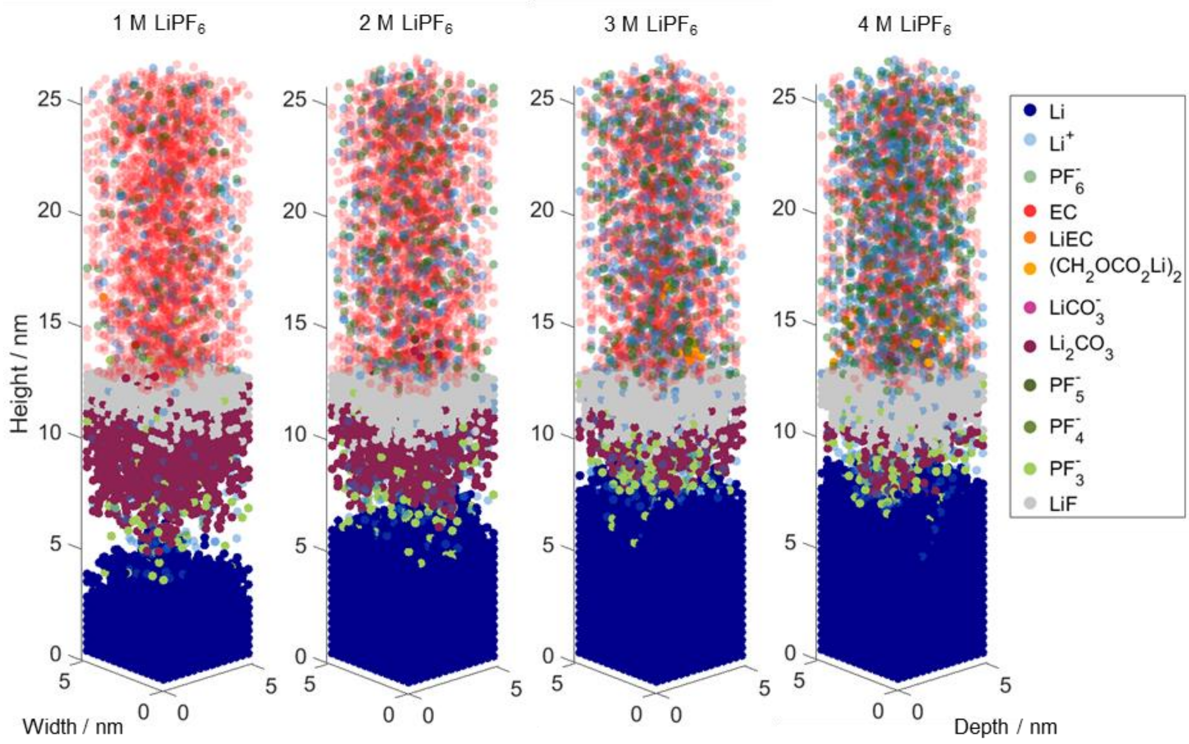

Supplementary Figure 4 Resulting species distribution in kMC boxes for a variation of the conductive salt concentrations after 1µs. Source data are provided as a Source Data file.

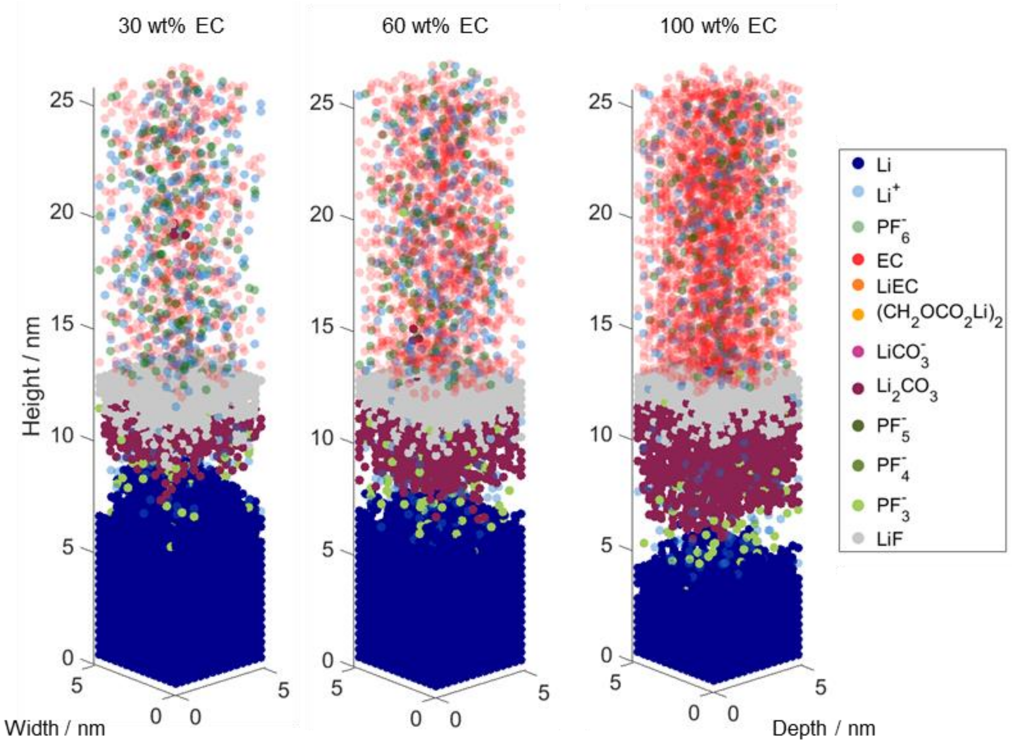

Supplementary Figure 5 Resulting species distribution in kMC boxes for a variation of the EC weight fractions after 1µs. Source data are provided as a Source Data file.

7. Effect of the electrical double layer on the SEI formation in our model:

The electrical double layer (EDL) has multiple effects on the formation of the SEI on lithium metal. First of all, it influences the ion distribution in the liquid electrolyte and hence the local concentration of salt and Li^+-ions as well as charged intermediate species. Thereby, it impacts the availability of these species for reduction reactions. At the same time, the EDL is affected by the formed SEI, and therefore it is constantly changing during SEI formation[9]. Once stable SEI layers have formed, we expect double layers to form at the interface between electrode and SEI, as it is an ion conducting layer, and between SEI and liquid electrolyte -which has a different ion concentration as the SEI. Such a model with two double layers was able to reproduce experiments in Li-ion batteries including the two time semicircles in impedance spectra[10]. In addition, typical times for EDL charge/discharge in batteries are in the order of milliseconds to seconds[11], which is far beyond the here considered time scale.

Still, in our model the effect of the EDL is considered in various ways. First of all, since the lithium metal atoms from the anode are oxidized during the simulation, there are many positively charged Li+-ions available close to the surface. Further, we account for local electroneutrality by considering the charge of the direct environment in the rate calculation of the transport of charged species (Equation 6). Third, the double layer capacity is included in our continuum model according to Equation 14. These implemented phenomena result in the accumulation of positive charges close to the lithium metal surface within the first 100 ns as can be seen in Supplementary Figure 3. This vanishes after the first inorganic SEI has formed after the first 1µs. Hence, on larger time scales a more elaborate consideration of the EDL including the effect of the SEI might be required. Since this is out of the scope of the present study, this method development is of high interest for future investigations.

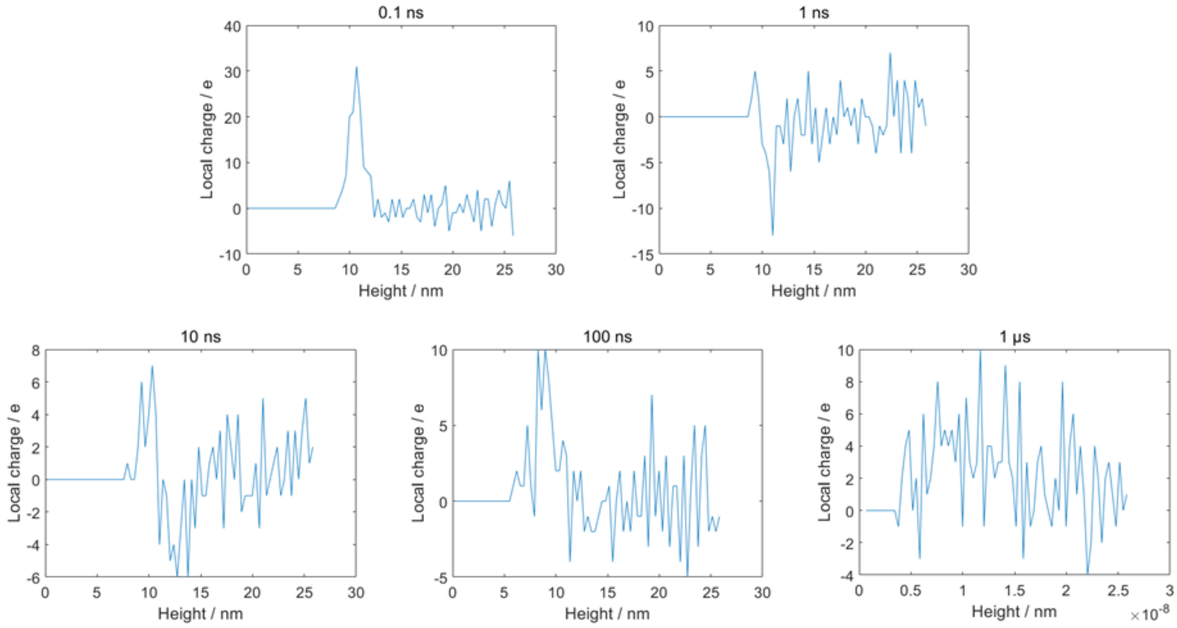

Supplementary Figure 6 Charge accumulation during SEI formation as function of height and time, showing the build-up of a double layer at the Li surface, which is subsequently consumed by SEI formation. Source data are provided as a Source Data file.

Supplementary Table 2 Summary of number of initially present EC molecules ($n_{EC,init}$) and salt molecules ($n_{salt,init}$) in the electron transport zone, number of overall consumed EC molecules ($n_{EC,total}$) and salt molecules ($n_{salt,total}$) and the respective ratio of initially present and overall consumed molecules for all conducted simulations.

Simulation	$n_{EC,init}$	$n_{EC,total}$	$\frac{n_{EC,init}}{n_{EC,total}}$	$n_{salt,init}$	$n_{salt,total}$	$\frac{n_{salt,init}}{n_{salt,total}}$
1M $LiPF_6$	505	4354	0.116	38	329	0.116
2M $LiPF_6$	431	2933	0.147	78	353	0.221
3M $LiPF_6$	420	2128	0.197	121	396	0.306
4M $LiPF_6$	352	1825	0.193	167	436	0.383
30 wt-% EC	147	1973	0.075	48	335	0.143
60 wt% EC	264	2639	0.1	51	324	0.157
100 wt% EC	534	4044	0.132	39	330	0.118

8. Details on DFT results

Supplementary Table 3 Electronic energetics of structures in Table 1, computed at B3PW91/6-311G(3df) level of theory with solvation model based on density (SMD) implemented. C, M represents the charge and multiplicity of the corresponding species, Pot. E. describes the potential energy, H, S and G stand for the enthalpy, entropy and free energy, respectively. The G1M column stands for the free energy value converted from 1 atm to 1 M at standard condition. All energy values are in kcal/mol. The atomic cartesian coordinates of all structures are provided in the Source Data file.

Structure	C, M	Pot. E.	H	S	G	G1M
Li	0,2	-7.48424	-7.48188	33.175	-7.49764	-7.49462
Li^+	1,1	-7.42082	-7.41846	31.798	-7.43357	-7.43055
EC	0,1	-342.389	-342.309	69.277	-342.342	-342.339
$[EC-Li]^+$	1,1	-349.838	-349.755	75.223	-349.791	-349.788
$[EC-Li]^+ + e^-$	0,2	-349.894	-349.811	80.678	-349.849	-349.846
$[EC-Li]^0$	0,2	-349.911	-349.829	80.333	-349.867	-349.864
TS-R2	0,2	-349.888	-349.81	80.947	-349.848	-349.845
LiEC	0,2	-349.948	-349.87	88.085	-349.912	-349.909
C_2H_4	0,1	-78.5769	-78.522	52.297	-78.5468	-78.5438
TS-R3	0,3	-699.911	-699.753	132.139	-699.816	-699.812
LiEDC	0,1	-621.421	-621.313	112.859	-621.366	-621.363
$[LiEC]^-$	-1,1	-350.026	-349.952	75.253	-349.987	-349.984
TS-R4	-1,1	-350.121	-350.044	92.141	-350.088	-350.085
$LiCO_3^-$	-1,1	-271.502	-271.482	63.81	-271.513	-271.51
Li_2CO_3	0,1	-279.02	-278.995	74.181	-279.03	-279.027

Supplementary References

1. Andersen, M., Panosetti, C. & Reuter, K. A practical guide to surface kinetic Monte Carlo simulations. *Front. Chem.* **7**, 1–24 (2019).
2. Gerasimov, M. *et al.* Species Distribution During Solid Electrolyte Interphase Formation on Lithium Using MD/DFT-Parameterized Kinetic Monte Carlo Simulations. *J. Phys. Chem. C* **127**, 4872–4886 (2023).
3. Neuhaus, J., Bellaire, D., Kohns, M., von Harbou, E. & Hasse, H. Self-Diffusion Coefficients in Solutions of Lithium Bis(fluorosulfonyl)imide with Dimethyl Carbonate and Ethylene Carbonate. *Chemie-Ingenieur-Technik* **91**, 1633–1639 (2019).
4. Röder, F., Braatz, R. D. & Krewer, U. Direct coupling of continuum and kinetic Monte Carlo models for multiscale simulation of electrochemical systems. *Comput. Chem. Eng.* **121**, 722–735 (2019).
5. von Kolzenberg, L., Latz, A. & Horstmann, B. Solid-Electrolyte Interphase During Battery Cycling: Theory of Growth Regimes. 1–11 (2020).
6. Wrodnigg, G. H., Besenhard, J. O. & Winter, M. Cyclic and acyclic sulfites: New solvents and electrolyte additives for lithium ion batteries with graphitic anodes? *J. Power Sources* **97–98**, 592–594 (2001).
7. Schulze, T. P. Kinetic Monte Carlo simulations with minimal searching. *Phys. Rev. E - Stat. Physics, Plasmas, Fluids, Relat. Interdiscip. Top.* **65**, 1–3 (2002).
8. Ospina-Acevedo, F., Guo, N. & Balbuena, P. B. Lithium oxidation and electrolyte decomposition at Li-metal/liquid electrolyte interfaces. *J. Mater. Chem. A* **8**, 17036–17055 (2020).
9. Christensen, J. *et al.* Computational Exploration of the Li-Electrode|Electrolyte Interface in the Presence of a Nanometer Thick Solid-Electrolyte Interphase Layer. *J. Electrochem. Energy Convers. Storage* **158**, 1512–1517 (2016).
10. Witt, D., Röder, F. & Krewer, U. Analysis of Lithium-Ion Battery State and Degradation via Physicochemical Cell and SEI Modeling. *Batter. Supercaps* **5**, e202200067 (2022).
11. Krewer, U. *et al.* Review—Dynamic Models of Li-Ion Batteries for Diagnosis and Operation: A Review and Perspective. *J. Electrochem. Soc.* **165**, A3656–A3673 (2018).
